# How introduction of automated insulin delivery systems may influence psychosocial outcomes in adults with type 1 diabetes: Findings from the first investigation with the Omnipod^®^ 5 System

**DOI:** 10.1016/j.diabres.2022.109998

**Published:** 2022-07-16

**Authors:** William H. Polonsky, Korey K. Hood, Carol J. Levy, Sarah A. MacLeish, Irl B. Hirsch, Sue A. Brown, Bruce W. Bode, Anders L. Carlson, Viral N. Shah, Ruth S. Weinstock, Anuj Bhargava, Thomas C. Jones, Grazia Aleppo, Sanjeev N. Mehta, Lori M. Laffel, Gregory P. Forlenza, Jennifer L. Sherr, Lauren M. Huyett, Todd E. Vienneau, Trang T. Ly, Omnipod 5 Research Group

**Affiliations:** a Behavioral Diabetes Institute, 5230 Carrol Canyon Road Ste 208, San Diego, CA 92121, United States; b University of California San Diego, 9500 Gilman Dr, La Jolla, CA 92093, United States; c Department of Pediatrics, Psychiatry & Behavioral Sciences, Stanford Diabetes Research Center, Stanford University School of Medicine, 279 Campus Drive, B300, Stanford, CA 94305, United States; d Icahn School of Medicine at Mount Sinai, 1 Gustave L. Levy Pl, New York, NY 10029, United States; e University Hospitals Cleveland Medical Center, Rainbow Babies and Children’s Hospital, 11100 Euclid Ave, Cleveland, OH 44106, United States; f Department of Medicine, University of Washington, 750 Republican Street, Building F, Floor 3, Seattle, WA 98109, United States; g Division of Endocrinology, Center for Diabetes Technology, University of Virginia, 560 Ray C Hunt Dr, Charlottesville, VA 22903, United States; h Atlanta Diabetes Associates, 1800 Howell Mill Rd #450, Atlanta, GA 30318, United States; i International Diabetes Center, Park Nicollet, HealthPartners, 3800 Park Nicollet Blvd, Minneapolis, MN 55415, United States; j Barbara Davis Center for Diabetes, University of Colorado Anschutz Medical Campus, 1775 Aurora Ct #A140, Aurora, CO 80045, United States; k Department of Medicine, SUNY Upstate Medical University, 750 E Adams St, Syracuse, NY 13210, United States; l Iowa Diabetes Research, 1031 Office Park Rd Suite #2, West Des Moines, IA 50265, United States; m Department of Research, East Coast Institute for Research at The Jones Center, 265 Sheraton Blvd, Macon, GA 31210, United States; n Feinberg School of Medicine, Northwestern University, 645 N Michigan Ave Ste 530, Chicago, IL 60611, United States; o Joslin Diabetes Center, Harvard Medical School, One Joslin Place, Boston, MA 02215, United States; p Department of Pediatrics, Yale School of Medicine, 333 Cedar St, New Haven, CT 06510, United States; q Insulet Corporation, 100 Nagog Park, Acton, MA 01720, United States

**Keywords:** Automated insulin delivery, Psychosocial outcomes, Type 1 diabetes, Closed-loop system

## Abstract

**Aims:**

To evaluate psychosocial outcomes for adults with type 1 diabetes (T1D) using the tubeless Omnipod^®^ 5 Automated Insulin Delivery (AID) System.

**Methods:**

A single-arm, multicenter (across the United States), prospective safety and efficacy study of the tubeless AID system included 115 adults with T1D. Participants aged 18–70 years completed questionnaires assessing psychosocial outcomes – diabetes distress (T1-DDS), hypoglycemic confidence (HCS), well-being (WHO-5), sleep quality (PSQI), insulin delivery satisfaction (IDSS), diabetes treatment satisfaction (DTSQ), and system usability (SUS) – before and after 3 months of AID use. Associations among participant characteristics, psychosocial measures and glycemic outcomes were evaluated using linear regression analyses.

**Results:**

Adults using the tubeless AID system demonstrated improvements in diabetes-specific psychosocial measures, including diabetes distress, hypoglycemic confidence, insulin delivery satisfaction, diabetes treatment satisfaction, and system usability after 3 months (all P < 0.001). No changes in general well-being or sleep quality were observed. The psychosocial outcomes assessed were not consistently associated with baseline participant characteristics (i.e., age, sex, diabetes duration, glycemic outcomes including percent time in range 70–180 mg/dL, percent time below range < 70 mg/dL, hemoglobin A1c, or insulin regimen).

**Conclusions:**

Use of the Omnipod 5 AID system was associated with significant improvements in diabetes-related psychosocial outcomes for adults with T1D.

## 1. Introduction

For persons with type 1 diabetes (T1D), successful management requires ongoing time, effort, and attention, which can take a toll on psychosocial health [1]. In addition to the common stressors of adult life – maintaining family needs, work-life balance, social demands, and attention to one’s general health, diet, and exercise – people with T1D must manage the additional worries, fears, and concerns specific to their diabetes regimen and self-management goals. These include day-to-day concerns about glucose levels as well as concerns about long-term health and the risk of microvascular and macrovascular complications. Due to these factors, T1D is often associated with significant diabetes-related emotional distress (“diabetes distress”) [2] and, more broadly, impaired quality of life (QOL) [3,4]. Furthermore, diabetes distress can contribute to poor glycemic control [5]. There is a strong and pressing need for new technologies, such as automated insulin delivery (AID) systems, which may reduce the behavioral burden of diabetes self-management while improving health outcomes.

The Omnipod^®^ 5 AID system is an on-body system consisting of a tubeless insulin pump (Pod) with a built-in algorithm that communicates directly with a continuous glucose monitor (CGM) and a smartphone with the Omnipod 5 App to inform automated insulin dosing [6,7]. The system has been shown to be safe and effective in the management of children, adolescents and adults with T1D, with minimal episodes of severe hypoglycemia and diabetic ketoacidosis, and with significant improvements in glycemic outcomes, including decreased hemoglobin A1c (HbA1c) and increased percent time in range (TIR, % 70–180 mg/dL) [8].

In addition to improving glycemic outcomes, it is important that AID systems improve psychosocial metrics to support broad adoption and long-term use of these technologies. While several AID clinical trials have documented a positive impact on diabetes distress after using AID [9,10], there remains a need to advance our understanding of the broader psychosocial outcomes associated with AID systems use, including a tubeless AID system which offers unique features. We investigated change in these diabetes-specific and general psychosocial metrics in adults with T1D over the 3-month period during the Omnipod 5 pivotal trial. Psychosocial measures included diabetes distress, confidence managing hypoglycemia, insulin delivery satisfaction, diabetes treatment satisfaction, usability, well-being, and sleep quality.

## 2. Subjects, materials and methods

### 2.1. The Omnipod 5 pivotal trial

A single-arm, prospective, multi-center clinical study enrolling 241 participants was conducted at 17 institutions in the United States. Details of the study design and primary outcomes have been published previously [8]. Key inclusion criteria were ages 6.0–70.0 years, a point-of-care screening HbA1c < 10.0 % (86 mmol/mol), and a diagnosis of T1D for a minimum of 6 months. Exclusion criteria included history of severe hypoglycemia or diabetic ketoacidosis (unrelated to intercurrent illness, infusion set failure, or initial diagnosis) in the past 6 months, or diagnosed with anorexia nervosa or bulimia, acute or chronic kidney disease, sickle cell disease, or hemophilia or any other bleeding disorders. Those with inability to tolerate adhesive tape and those using non-insulin anti-diabetes medications other than metformin were excluded. After screening, CGM data were collected for two weeks with the study sensor (Dexcom G6) while participants used their usual therapy (‘standard therapy’ (ST) phase). The study sensor was blinded during the ST phase for participants not using the Dexcom G6 CGM as part of their usual therapy regimen (50 of the 241 participants). Those using the Dexcom G6 CGM as part of their usual therapy regimen were allowed to provide retrospective data from their Dexcom G6 use for 14 days of the last 30 days prior to AID initiation to serve as their ST phase.

After collection of baseline measures, the participants were trained to use the investigational AID system consisting of a tubeless, on-body insulin pump (Pod) with embedded model predictive control algorithm that communicates directly with an interoperable glucose sensor (Dexcom G6^®^, Dexcom Inc., San Diego, CA), and a smartphone application (Omnipod 5 app) on a locked-down Android phone. Additional system function details were previously published [6–8]. Between 19 December 2019 and 28 February 2020, participants were enrolled and entered the AID phase. Participants used the system for 3 months during which 9 follow-up study visits occurred by phone or in person. AID use was paused study-wide as a precaution from 28 February 2020 to 4 June 2020 because of a software anomaly with the potential to impact insulin delivery as a result of erroneous system inputs in certain uncommon circumstances. There were no adverse events associated with the anomaly. Once the software update was implemented, participants resumed AID. The last visit of the study occurred on 18 September 2020. The clinical study protocol was approved by relevant local review boards and the central Institutional Review Board. The trial was registered through ClinicalTrials.gov (NCT04196140).

### 2.2. Participants

Two cohorts of participants were enrolled: children (6–13.9 years) and adolescents and adults (14–70 years); however, specific psychosocial measures were assessed according to the following group assignments: children (6–11.9 years), teens (12–17.9 years), and adults (18–70 years). This report evaluated the adult cohort (N = 115), all of whom provided written informed consent.

### 2.3. Assessment of psychosocial outcomes

Participants completed questionnaires assessing overall well-being (WHO-5), sleep quality (PSQI), diabetes distress (T1-DDS), and hypoglycemia confidence (HCS), as well as diabetes treatment satisfaction (DTSQ), insulin delivery device satisfaction (IDSS), and system usability (SUS) as detailed below. Questionnaires were completed before starting the AID system, in reference to the participants’ standard therapy, and again based on the AID system following 3 months of use (“follow-up”). The questionnaires given at the two timepoints were identical, except for the Diabetes Treatment Satisfaction Questionnaire (DTSQ): the DTSQ-status (DTSQs) questionnaire was given at baseline, while the DTSQ-change (DTSQc) questionnaire was given at follow-up. At follow-up, participants also completed a free response questionnaire about what they liked and disliked about the investigational system; responses were summarized according to general themes. All questionnaires were completed online, except for the DTSQ which was administered by paper. This study assessing the various psychosocial outcomes was exploratory, with no pre-specified primary or secondary outcomes.

#### 2.3.1. Type 1 Diabetes Distress Scale

The Type 1 Diabetes Distress Scale (T1-DDS) measures diabetes distress over the past month and includes seven subscales: powerlessness, management distress, hypoglycemia distress, negative social perceptions, eating distress, physician distress, and friend/family distress [2,11]. It consists of 28 items individually scored on a 6-point scale from 1 (‘Not a Problem’) to 6 (‘A Very Serious Problem’). An overall mean score < 2.0 indicates little or no distress, 2.0 to 2.9 indicates moderate distress, and ≥ 3.0 indicates high distress. Similarly, any overall or subscale mean score>2.0 is considered clinically significant [12].

#### 2.3.2. Hypoglycemia Confidence Scale

The Hypoglycemia Confidence Scale (HCS) assesses the degree to which people with diabetes feel able, secure, and comfortable about their ability to avoid or address hypoglycemia-related problems [13]. Each of the 9 items on the HCS is rated on a 4-point scale from 1 (‘Not Confident at All’) to 4 (‘Very Confident’). A higher mean score indicates more confidence in managing hypoglycemia-related issues with a score ≥ 3 indicating relatively high confidence [13].

#### 2.3.3. World Health Organization 5 Well-Being Index

The World Health Organization 5 Well-Being Index (WHO-5) assesses overall well-being over the last two weeks consisting of 5 items rated on a 6-point (0 to 5) Likert scale with higher scores defining higher well-being [14]. Individual item scores are summed and multiplied by four to obtain a total percentage score with 0 representing the lowest possible well-being and 100 representing the best well-being. A percentage score ≤ 50 suggests low mood [15].

#### 2.3.4. Pittsburgh Sleep Quality Index

The Pittsburgh Sleep Quality Index (PSQI) measures sleep disturbance and typical sleep habits over the past month [16]. It differentiates poor sleep from good sleep by measuring 7 components: sleep quality, sleep latency, sleep duration, habitual sleep efficiency, sleep disturbances, use of sleep medication, and daytime dysfunction. The PSQI consists of 19 self-rated questions and 5 questions rated by the bed partner or roommate (if one was available). The 19 self-rated items are used to derive 7 component scores. These component scores range from 0 (better) to 3 (worse) and are then added together, resulting in a total score ranging from 0 (indicating no difficulty with sleep) to 21 (indicating severe difficulties with sleep). A total score ≥ 5 is considered an indicator of poor sleep quality.

#### 2.3.5. Insulin Device Satisfaction Survey (T1)

The Insulin Device Satisfaction Survey (IDSS, T1 version) assesses patient satisfaction with their insulin delivery device along three dimensions: effectiveness, burden, and inconvenience [17]. The survey’s 14 items are rated on a 5-point scale ranging from 1 (‘Strongly Disagree’) to 5 (‘Strongly Agree’). The three subscale scores are obtained by calculating the mean score for items associated with each subscale; the total score is based on the mean of all items with the responses to specific items for the burdensome and inconvenient subscales reverse-coded. For the overall scale and effectiveness subscale, a higher value indicates greater satisfaction, while for the burdensome and inconvenient subscales scores, a lower value defines greater satisfaction.

#### 2.3.6. Diabetes Treatment Satisfaction Questionnaire

The Diabetes Treatment Satisfaction Questionnaires (DTSQ) [18] are 6-item scales that measure satisfaction with the diabetes treatment regimen. The DTSQ-status (DTSQs) version measures current satisfaction status [19] while the DTSQ-change (DTSQc) asks participants to evaluate how their satisfaction has changed with their current treatment as compared with their previous treatment [20,21]. The DTSQs items are scored from 0 to 6 and are summed with a possible total score range of 0 to 36, with 36 being the optimal score with the highest satisfaction. The DTSQc items are scored from −3 to 3 and are summed with possible total score −18 to 18; a positive DTSQc score (>0) indicates greater satisfaction with the current treatment in comparison to the prior treatment. The DTSQs and DTSQc also include two standalone items to self-report frequency of unacceptable low and high blood glucose.

#### 2.3.7. System Usability Scale

The System Usability Scale (SUS) is a validated questionnaire to assess perceived usability of technologies across industries [22,23], including diabetes technology [9,24]. The SUS is a 10-item questionnaire based on a 5-point Likert scale ranging from 1 (‘Strongly Disagree’) to 5 (‘Strongly Agree’). The items assess if users find the product intuitive and easy to use, how confident they feel about using the product, whether they think the product could be learned without technical support, and whether they think most people could learn to use it quickly. Answers are summed, with negatively-worded questions reverse-coded, and then rescaled to give an overall usability index from 0 to 100. A higher score indicates a greater level of usability; a score of 68 is considered average.

### 2.4. Statistical methods

Psychosocial outcome scores were compared between baseline (in reference to participants’ standard therapy) and after 3 months of AID system use, including total scores and subscales scores. Four participants withdrew or exited early from the study partway through the AID phase but still completed the follow-up questionnaires in reference to the AID system upon exit and were included in the analysis. The distribution of change in each score was tested for normality using the Shapiro-Wilk test. Data for all questionnaires were not normally distributed so the unadjusted Wilcoxon signed rank test was used. No adjustments for multiple comparisons were made. The magnitude of the treatment effect on change in questionnaire score, i.e., the effect size, was calculated using Cohen’s *d*, defined as the mean of the change divided by the standard deviation of the change [25]. A commonly used interpretation of Cohen’s *d* is small (*d =* 0.2), medium (*d =* 0.5), and large (*d =* 0.8) [26]. Missing data were minimal and imputation did not lead to meaningfully different results, therefore, only the results of the analysis without imputation are presented. Further analysis of differences in changes in psychosocial outcomes between forms of therapy at entry (MDI [multiple daily injections], tubed pumps, or tubeless pump) and between HbA1c at baseline categories (<7% or ≥ 7 % [53 mmol/mol)]) were evaluated using t-tests.

Exploratory analyses using linear regression models were designed to assess the effects of various predictors on the change in psychosocial outcomes; for DSTQ, the DTSQc score was modeled. For consistency in interpreting the regression results, the change in score for each questionnaire was calculated such that a positive change indicated an improvement. For each questionnaire, a multiple linear regression model was used to assess the effects of 6 participant baseline factors (baseline questionnaire score, age, diabetes duration, sex, percent time below range (TBR, % <70 mg/dL), and percent TIR (% 70–180 mg/dL)). Single linear regression models were used to assess the effects of changes in glycemic outcomes, including both percent TBR and percent TIR, on changes in questionnaire score. No adjustments for multiplicity were made to the models. All p-values were considered significant by a two-sided value of 0.05. Analysis was performed using SAS version 9.4 and GraphPad Prism version 9.2.

## 3. Results

### 3.1. Participant characteristics

A total of 115 adult participants initiated use of the AID system in the trial (Table 1). Fifty-four participants (47.0 %) were already meeting the American Diabetes Association (ADA) HbA1c target of < 7.0 % [53 mmol/mol] and 52 participants (45.2 %) were meeting the consensus TIR 70–180 mg/dL target of > 70 % at baseline. Different types of standard therapy used by the participants included tubeless pump (n = 62, 53.9 %), MDI (n = 18, 15.7 %), or tubed pump (n = 35, 30.4 %). There were 17 people (14.8 %) using tubed pump models with hybrid closed-loop (HCL) capabilities, 11 (9.6 %) using tubed pump models with predictive low glucose suspend (PLGS) capabilities, and 2 (1.7 %) using tubed pump models with low glucose suspend (LGS) capabilities; however, information was not recorded about whether participants were actively using these capabilities with a connected CGM during the standard therapy phase. Two participants (1.7 %) reported using a do-it-yourself AID system (one with a tubeless pump). Most (98 %) had previous experience using CGM. Ninety-seven percent (n = 111) of participants completed the full study. Reasons for early withdrawal included dissatisfaction with blood glucose control, preference for previous therapy, frustration with CGM and Pod connectivity, and moving away from the clinical site.

### 3.2. Psychosocial outcomes

Psychosocial outcomes are summarized in Table 2. Significant improvement was observed in both diabetes distress (T1-DDS, P < 0.0001) and hypoglycemia confidence (HCS, P = 0.0002) after 3 months of AID use. Of the seven T1-DDS subscales, participants reported significant benefits in five: reductions in powerlessness (P = 0.001), management distress (P = 0.0004), hypoglycemia distress (P < 0.0001), eating distress (P = 0.0003), and physician distress (P = 0.04). Baseline scores indicated participants had relatively low diabetes distress (T1-DDS < 2, mean: 1.64 ± 0.51) that decreased further over the 3-month period. No change was observed in well-being (WHO-5) or sleep quality (PSQI).

Significant improvements in treatment satisfaction and usability with the AID system compared with the prior (pre-study) treatment were observed across all three measures: greater insulin delivery satisfaction (IDSS, P = 0.0007), greater overall treatment satisfaction (DTSQc, P < 0.0001), and better system usability (SUS, P < 0.0001). Of note, of the three IDSS subscales, participants reported significant reductions in the burden (P = 0.004) and inconvenience (P = 0.002) of the insulin delivery device, but not system effectiveness (P = 0.07). The DTSQs had a mean of 28.4 ± 4.8 at baseline, indicating high initial treatment satisfaction that rose even higher by the end of the trial. The two DTSQc items concerning self-reported frequency of unacceptable low and high blood glucose also improved (both P < 0.001). Observed changes in psychosocial outcomes did not differ between groups according to prior therapy type (MDI, tubed pumps, or tubeless pump users) or baseline HbA1c (<7.0 % and ≥ 7.0 % [53 mmol/mol]) (Table S1 and S2, respectively).

Answers to the free response questionnaire about participants’ likes and dislikes related to the system were categorized and are displayed in Table 3. Common positive themes included: improved glucose management, improved nighttime control, favoring automated insulin delivery, hypoglycemia prevention, and reduced cognitive burden. In contrast, commonly cited areas for improvement included: Pod connectivity, algorithm performance, the need to wear the CGM and Pod on the same area of the body, or within same line of sight, and alarm sound/volume.

### 3.3. Multiple and single linear regression modelling

Improvements in most psychosocial outcomes were not associated with participant age, diabetes duration, sex, or baseline glycemic control (either % TBR or % TIR) (Table 4). There were two exceptions concerning satisfaction, which was associated with age and baseline glycemic control. Specifically, improvement in IDSS was associated with older age and greater % TBR at baseline (P = 0.04 and P = 0.01, respectively), and improvement in DTSQc was associated with older age and lower % TIR at baseline (P = 0.03 and P = 0.008, respectively). Furthermore, there was no consistent association between changes in either of the two major glycemic indices (% TBR and % TIR) and psychosocial outcomes; however, the IDSS was again an exception, with improvement in satisfaction being associated with a reduction in % TBR (P = 0.02).

## 4. Discussion

In this 3-month single-arm trial, we found that adults with T1D initiating therapy with the Omnipod 5 AID System experienced significant improvement in diabetes distress and hypoglycemia confidence. Furthermore, compared with their prior treatment method, study participants reported significant improvement in all measures of treatment satisfaction and system usability. Despite significant improvements in important diabetes-related psychosocial outcomes, there was no change in general measures of well-being and sleep quality. These results are consistent with findings from previous diabetes device studies, where improvements in diabetes-specific, but not general, psychosocial outcomes were more consistently observed [27,28]. We hypothesize that general measures of well-being may not have adequate sensitivity to detect changes in disease-specific psychosocial status in evaluations of AID systems, but this warrants additional investigation.

To our knowledge, this is the first report of psychosocial outcomes with a tubeless AID system. It is important to note that this group of adults with T1D were already experiencing positive outcomes with their prior therapy, with relatively low HbA1c and favorable psychosocial outcomes at the start of the study. A sizeable fraction of participants were already meeting the ADA and international consensus targets for glycemia at baseline (47 % with HbA1c < 7 % [53 mmol/mol], mean entry HbA1c for the entire study sample: 7.1 ± 0.9 % [54 ± 9.8 mmol/mol]; 45 % with TIR > 70 %, mean entry TIR for the entire study sample: 65.2 ± 17.0 %), and baseline psychosocial measures pointed to relatively low levels of diabetes distress (T1-DDS < 2, mean: 1.64 ± 0.51), high hypoglycemic confidence (HCS > 3, mean: 3.52 ± 0.45), and high usability with previous therapy (SUS > 68, mean: 75.9 ± 16.8); yet, improvements in each of these outcomes were still observed after three months of tubeless AID use. These results indicate that use of the Omnipod 5 System in the study was associated with additional benefits beyond those attained with prior therapy, suggesting that new technologies can offer adults with T1D more than what was previously thought to be achievable.

In general, the observed changes in psychosocial outcomes were not predicted by any of the baseline characteristics, suggesting that no matter the age, diabetes duration, gender, or baseline glycemic status, participants saw similar improvements in psychosocial outcomes. Furthermore, improvement in psychosocial outcomes were observed regardless of baseline HbA1c or prior insulin delivery system. Likewise, we found that change in psychosocial outcomes over time was generally unrelated to change in any of the glycemic indices. The one notable exception was that improvement in insulin delivery system satisfaction was associated with a reduction in % TBR. This result is consistent with the positive testimonials received from participants, where hypoglycemia benefits with tubeless AID system use were commonly mentioned responses. We note caution when interpreting these results as these were exploratory analyses without adjustments made for multiplicity, with the possibility of findings made by chance.

Psychosocial outcomes have been evaluated in other AID clinical trials. Consistent with our own positive findings regarding diabetes distress, in a 6-month trial of 168 participants, ages 14–71 years, randomized to a tubed AID system (N = 112) versus sensor augmented pump (SAP) (N = 56), Kudva and colleagues found that diabetes distress (T1-DDS) dropped significantly for the AID participants (P = 0.04), but not the SAP participants [9]. Similarly, in a small cohort of 14 adults and 15 adolescents in a clinical trial of an investigational version of another tubed AID system, diabetes distress (in this case, using only the T1-DDS management subscale) was reduced significantly by study end [10]. Hypoglycemia confidence (HCS) did not improve in a crossover trial (investigational advanced hybrid closed loop vs commercially available system, both using tubed pumps) with 113 adolescents and young adults, ages 14–29 years [29].

Strengths of this study include a relatively wide range of adult participants with respect to age and prior therapy including MDI, continuous subcutaneous insulin infusion (CSII), and AID users. The study also assessed a relatively broad range of psychometric instruments to obtain a more comprehensive understanding of psychosocial outcomes, including diabetes distress and hypoglycemia confidence as well as system usability and satisfaction with the tubeless AID system.

Limitations for this study include a single-arm design without a randomized control group comparison; thus, we cannot be certain that the observed psychosocial benefits resulted directly from the AID intervention. Secondly, the study was limited to a 3-month period with frequent interactions with the clinical team, so we do not know whether the positive impact on psychosocial measures will be maintained over time and in a real-world environment. Thirdly, a majority of study participants were non-Hispanic white (85.2 %), many of whom at enrollment were already achieving near-goal HbA1c, which limits generalizability and our ability to understand how psychosocial outcomes may differ across racial and ethnic groups as well as patients with broader ranges of glycemic control. However, the percentage of non-Hispanic white participants in our T1D sample is similar to that reported by the T1D Exchange Registry based on data from patients of all ages at 81 US-based endocrinology practices across 35 states (82 %) [30]. In addition, the exclusion of participants with HbA1c > 10 % for this study, who may have a greater potential for improvements, introduces a selection bias which also limits generalizability of the study’s results. Many of the improvements reported could have resulted from use of an AID system in general, and further research is needed to explain what benefits result from a tubeless AID system specifically. Lastly, income and education level of participants were not collected, so these study population characteristics were unknown. A postmarket study is planned to collect this type of information and evaluate the system in a larger sample that is more representative of the T1D population. Within these constraints, however, we conclude that use of the Omnipod 5 AID System was associated with significant improvements in diabetes distress, usability, and satisfaction in this sample of adults with T1D.

When assessing options for diabetes management, it is important that providers and patients not only consider safety and effectiveness of various treatment methods, but also evaluate ease-of-use and potential for such systems to relieve some of the daily self-care challenges and worries that people with T1D face every day. The results of this study demonstrate that the Omnipod 5 AID System may reduce the emotional and behavioral burdens of T1D, heighten the user’s confidence in avoiding or addressing hypoglycemia-related problems, and increase their satisfaction with insulin delivery and diabetes management, while also assuring perceptions of relative ease-of-use. Taken together, these results indicate that the Omnipod 5 AID System evaluated in this study could offer a valuable option to potentially relieve some of the emotionally and cognitively taxing aspects of diabetes management for adults with T1D.

### Questionnaire Copyright Statements

Diabetes Treatment Satisfaction Questionnaire status and change ^©^ Prof. Clare Bradley, can be accessed via www.healthpsychologyresearch.com, Health Psychology Research, 188 High Street, Egham, Surrey, TW20 9ED.

Pittsburgh Sleep Quality Index: A New Instrument for Psychiatric Practice and Research (Authors Daniel J. Buysse, Charles F. Reynolds III, Timothy H. Monk, Susan R. Berman, and David J Kupfer, ^©^ 1989 and 2010, University of Pittsburgh. All rights reserved.)

### The Omnipod 5 Research Group Members

A listing of the Omnipod 5 Research Groups sites with participants included in the psychosocial outcomes analysis for adults with the principal investigator (PI) and co-investigators (Co-I) noted.

#### Psychosocial Outcomes Investigators

William H Polonsky PhD CDCES, Behavioral Diabetes Institute and University of California San Diego, Korey K. Hood PhD, Stanford University, Sarah A. MacLeish DO, University Hospitals Cleveland Medical Center, Rainbow Babies and Children’s Hospital.

#### Icahn School of Medicine at Mount Sinai, New York, NY

Carol J. Levy MD CDCES (PI), David W. Lam MD (Co-I), Camilla Levister NP CDCES (Co-I), Grenye O’Malley MD (Co-I), Selassie Ogyaadu MD MPH, Dushyanthy Arasaratnam B. Med, Mitchell Plesser BS, Emily Nosova MD.

#### Department of Medicine, University of Washington, Seattle, WA

Irl B. Hirsch MD (PI), Subbulaxmi Trikudanathan MD MRCP MMSc, Nancy Sanborn ND CDE, Dori Khakpour CD RDN CDCES.

#### Division of Endocrinology, Center for Diabetes Technology, University of Virginia, Charlottesville, VA

Sue A. Brown MD (PI), Mary Voelmle FNP (Co-I), Emma Emory RN.

#### Atlanta Diabetes Associates, Atlanta GA

Bruce W. Bode MD (PI), Brooke Narron, Tricia Lopez.

#### International Diabetes Center, Park Nicollet, HealthPartners, and Park Nicollet Pediatric Endocrinology, Minneapolis, MN

Anders L. Carlson MD (PI), Amy B. Criego MD (PI), Richard M. Bergenstal MD (PI), Thomas Martens MD, Aimee Grieme RN CDCES, Jamie Hyatt RN BSN CDCES, Alina Punel BSN RN, Diane Whipple RN BSN CCRC CDCES.

#### Barbara Davis Center for Diabetes, University of Colorado Anschutz Medical Campus, Aurora, CO – Adult Clinic

Viral N. Shah MD (PI), Halis K. Akturk MD, Nicole Schneider, Hal Joseph (Co-I), Prakriti Joshee, Christie Beatson.

#### Sansum Diabetes Research Institute, Santa Barbara, CA

Mei Mei Church MS NP CDCES, Kristin Castorino DO, Molly Piper, Jimena Perez.

#### SUNY Upstate Medical University, Syracuse NY

Ruth S. Weinstock MD PhD (PI), Suzan Bzdick RN CDCES CCRC, David W. Hansen MD MPH (PI), Sheri L. Stone RN MSN NP-C.

#### Department of Research, Iowa Diabetes Research, West Des Moines, IA

Anuj Bhargava MD (PI), Lisa Borg CMA CCRC.

#### East Coast Institute for Research at The Jones Center, Macon, GA

Thomas C. Jones MD (PI), Barry Russel Johns MD, Ashwini Gore MD, Leslie Harvill PA-C, Kayla Merritt NP-C, Jennifer Stanfield, Jennifer Sheldon NP-C, Lisa Hichkad PA-C, Erica Burnett NP-C, Alicia Castelot RMA CCRP, Lindsay Bounds, Kaitlyn Preston, Rebecca Goldfaden PharmD CCRP.

#### Feinberg School of Medicine, Northwestern University, Chicago, IL

Grazia Aleppo MD (PI), Jelena Kravarusic MD PhD (Co-I), Anupam Bansal MD, Candice Fulkerson RN.

#### Joslin Diabetes Center, Harvard Medical School, Boston, MA

Sanjeev N. Mehta MD MPH (PI), Lori M. Laffel MD MPH (Co-PI), Lindsay Roethke, Margaret Fisher, Rebecca Ortiz La Banca PhD RN, Lisa Volkening MA CCRP, Louise Ambler-Osborn PNP, Christine Turcotte PNP, Emily F. Freiner FNP.

#### Barbara Davis Center for Diabetes, University of Colorado Anschutz Medical Campus, Aurora, CO – Pediatric Clinic

Gregory P. Forlenza MD (PI), R. Paul Wadwa MD (Co-I), Robert Slover MD (Co-I), Erin Cobry MD (Co-I), Laurel H Messer RN PhD, Cari Berget RN MPH CDE, Susan McCoy RN, Luke Geiser BS.

#### Department of Pediatrics, Yale School of Medicine, New Haven, CT

Jennifer L. Sherr MD PhD (PI), Michelle Van Name MD (Co-I), Michelle Brei DNP (Co-I), Melinda Zgorski BSN, Amy Steffen BSN, Lori Carria MS.

#### Labcorp, Burlington, NC

Kaisa Kivilaid MS, Krista Kleve MS, Matthew Partridge MS.

#### Insulet Corporation, Acton, MA

Trang T. Ly MBBS, Todd E. Vienneau BSc, Lauren M. Huyett PhD, Bonnie Dumais RN, Noel Schaeffer PhD, Armando Alvarez MS, Rachel E. Gurlin PhD.

## Supplementary Material

1

2

3

## Figures and Tables

**Table 1:** Characteristics of adult participants at baseline.

Characteristic	
**N**	115
**Age (years),** [range]^a^	39.3 ± 12.7 [18.2, 69.8]
**Duration of diabetes (years),** [range]	19.0 ± 11.7 [1.0, 51.0]
**Body mass index (kg/m**^**2**^**),** [range]^b^	27.0 ± 4.7 [19.0, 41.4]
**Female sex – no. (%)**	72 (62.6)
**Race/Ethnicity – no. (%)** ^c^	
White	104 (90.4)
Hispanic or Latino	6 (5.2)
Not Hispanic or Latino	98 (85.2)
Black or African American	5 (4.3)
Hispanic or Latino	1 (0.9)
Not Hispanic or Latino	4 (3.5)
Asian	2 (1.7)
American Indian or Alaska Native, White	1 (0.9)
American Indian or Alaska Native	3 (2.6)
Hispanic or Latino	3 (2.6)
Not Hispanic or Latino	–
**Time in Range (TIR) 70**–**180 mg/dL (%)**	65.2 ± 17.0
**TIR > 70 % – no. (%)**	52 (45.2)
**HbA1c (%)** [range], **(mmol/mol)** [range]	7.1 ± 0.9 [5.2, 9.8], (54 ± 9.8) [33, 84]
**HbA1c < 7.0 % [53 mmol/mol] – no. (%)**	54 (47.0)
**Previous** ^d^ **/current continuous glucose monitor use – no. (%)**	113 (98.3)
**Baseline standard therapy, MDI – no. (%)**	18 (15.7)
**Baseline standard therapy, pump – no. (%)**	97 (84.3)
Tubeless pump	62 (53.9)
Tubed pump	35 (30.4)
With low glucose suspend capabilities ^e^	2 (1.7)
With predictive low glucose suspend capabilities ^e^	11 (9.6)
With hybrid closed-loop capabilities ^e^	17 (14.8)

**Abbreviations:** HbA1c, hemoglobin A1c; TIR, time in range; MDI, multiple daily injections.

Data are mean ± SD and range [minimum, maximum] or n (%).

a Age was determined at the date of informed consent.

b Body-mass index is the weight in kilograms divided by the square of the height in meters.

c Race and ethnicity were reported by the participants and were not mutually exclusive.

d Previous use is defined as having used the device for any duration in the past.

e While the model of pump included these capabilities, information was not recorded about whether participants were actively using these features with a connected CGM during the standard therapy phase.

**Table 2:** Questionnaire and subscale scores (N = 115) at baseline and after 3 months of AID use.

Questionnaire	N	Score Range (Optimal Score)	Baseline	3 months of AID	Change^a^	P-value^b^	Cohen’s *d*
T1-DDS Overall	115	(1) to 6	1.64 ± 0.51 1.50 [1.32, 1.85]	1.48 ± 0.40 1.39 [1.21, 1.68]	−0.16 ± 0.39 −0.11 [−0.36, 0.04]	**<0.0001**	0.42
Powerlessness	115	(1) to 6	2.26 ± 0.98	2.02 ± 0.84	−0.24 ± 0.83	**0.0014**	0.29
Management Distress	114	(1) to 6	1.54 ± 0.60	1.38 ± 0.45	−0.16 ± 0.53	**0.0004**	0.30
Hypoglycemia Distress	115	(1) to 6	1.55 ± 0.64	1.33 ± 0.46	−0.22 ± 0.50	**<0.0001**	0.44
Negative Social Perceptions	115	(1) to 6	1.36 ± 0.61	1.28 ± 0.47	−0.09 ± 0.49	0.1297	0.18
Eating Distress	115	(1) to 6	1.97 ± 0.93	1.73 ± 0.76	−0.24 ± 0.66	**0.0003**	0.36
Physician Distress	115	(1) to 6	1.19 ± 0.52	1.12 ± 0.55	−0.07 ± 0.43	**0.0372**	0.16
Friend/Family Distress	115	(1) to 6	1.56 ± 0.76	1.43 ± 0.59	−0.13 ± 0.62	0.0772	0.21
HCS	115	1 to (4)	3.52 ± 0.45 3.67 [3.13, 3.89]	3.65 ± 0.37 3.78 [3.56, 3.89]	0.13 ± 0.35 0.11 [−0.11, 0.33]	**0.0002**	0.36
WHO-5	111	0 to (10 0)	69.4 ± 16.1 72.0 [60.0, 80.0]	69.1 ± 16.2 72.0 [60.0, 80.0]	−0.3 ± 13.7 0.0 [−8.0, 8.0]	0.7912	0.02
PSQI Total	91	(0) to 21	5.53 ± 2.75 5.00 [3.00, 7.00]	5.27 ± 2.70 5.00 [3.00, 7.00]	−0.25 ± 2.16 0.00 [−1.00, 1.00]	0.4217	0.12
IDSS Overall	115	1 to (5)	3.91 ± 0.49 3.86 [3.64, 4.29]	4.07 ± 0.58 4.21 [3.71, 4.50]	0.16 ± 0.68 0.21 [−0.14, 0.50]	**0.0007**	0.24
Effective	115	1 to (5)	4.22 ± 0.51	4.29 ± 0.71	0.07 ± 0.80	0.0743	0.09
Burdensome	115	(1) to 5	2.23 ± 0.59	2.06 ± 0.65	−0.17 ± 0.81	**0.0036**	0.21
Inconvenient	115	(1) to 5	2.31 ± 0.75	2.04 ± 0.76	−0.27 ± 0.93	**0.0024**	0.29
DTSQc^c^	114	−18 to (18)	–	12.6 ± 7.1 15.0 [11.0, 17.0]	–	**<0.0001**	–
Frequency of high BG	114	(−3) to 3	–	−0.5 ± 1.7	–	**0.0009**	–
Frequency of low BG	114	(−3) to 3	–	−1.6 ± 1.3	–	**<0.0001**	–
SUS	111	0 to (10 0)	75.9 ± 16.8 77.5 [65.0, 90.0]	83.8 ± 15.8 87.5 [75.0, 95.0]	7.9 ± 21.3 7.5 [0.0, 20.0]	**<0.0001**	0.37

Data are mean ± SD and median [IQR].

**Abbreviations:** AID, automated insulin delivery; T1-DDS, Type 1 Diabetes Distress Scale; HCS, Hypoglycemia Confidence Scale; WHO-5, World Health Organization Well-Being Index 5; PSQI, Pittsburgh Sleep Quality Index; IDSS, Insulin Delivery System Satisfaction; DTSQc, Diabetes Treatment Satisfaction Questionnaire – change; BG, blood glucose; SUS, System Usability Scale.

a Change is calculated as follow-up after 3 months of AID minus baseline score.

b Unadjusted two-sided Wilcoxon signed rank test.

c The DTSQc score is assessed as a change from 0.0 (no change in treatment satisfaction).

**Table 3:** Categorization of free response answers to questionnaire on likes and dislikes of the tubeless AID system, showing the most commonly-reported response categories for each question (reported by ≥ 10 % of participants).

Answer category	Number of users (%)	Representative quote
**1. What did you like most about [the study system]? (n = 114**^a^)		
Improved BG management	25 (22)	[Omnipod 5] was able to help me control my blood sugars while being a busy and active person. I liked that I was able to rely on the system to keep my blood sugars level.
Night time control	18 (16)	The security it gave me especially with regards to controlling low blood sugars overnight.
Automated insulin delivery	16 (14)	Where do I start? I love so much about [Omnipod 5]! What I like most is the interface between the two devices and the fact that the system is making adjustments to my dosage without any intervention from me.
Prevented lows	15 (13)	Even when I was not in HypoProtect^b^, the […] system did a great job of not letting me go low. I usually feel low symptoms around 75–80, and when I would start to feel symptoms with the […] system it would usually bring me back up to a comfortable range (without over-correcting and making me high) pretty quickly and without me having to eat or drink anything.
Reduced cognitive load	11 (10)	I appreciated it took care of the background noise of my diabetes management. I am currently in a traumatic life event and attention to my diabetes has suffered. The […] system helped keep me in target more often.
**2. What did you like least about [the system]? (N = 115**^a^)		
Pod connectivity	23 (20)	The connectivity issues….…occasionally it would look for the pod or cgm.
Algorithm performance	22 (19)	It felt like the system was heavily concentrated on preventing low blood sugars. If my blood sugar was high it seemed like it took too long to come down.
Wearing CGM and Pod on same area	17 (15)	I don’t like that the pod and transmitter need to be so close. This makes it hard to rotate sites.
Alarm volume/sound	15 (13)	I did not enjoy how loud and disruptive the alarm sounds were.

**Abbreviations:** BG, blood glucose; CGM, continuous glucose monitor.

a 114 and 115 participants responded to these questions, respectively; however, only response categories reported by ≥ 10 % of participants are shown. There were additional response categories reported by < 10 % of participants, which are not shown.

b In the investigational device during the study, users could activate the HypoProtect feature, which automatically raised the glucose target to 150 mg/dL and reduced insulin delivery. In the commercial system, this feature is renamed to “activity feature”.

**Table 4:** Estimated coefficients for multiple and single linear regression of change in questionnaire score.^a^

	Multiple Linear Regression						Single Linear Regression	
	Baseline Question-naire Score	Age (yr)	Diabetes Duration (yr)	Sex^b^	Baseline/ST % TBR <70 mg/dL	Baseline/ST % TIR 70–180 mg/dL	△ % TBR < 70 mg/dL^c^	△ % TIR 70–180 mg/dL^d^
△T1-DDS - Overall	**0.493** ***	0.000	0.001	0.021	−0.005	−0.001	−0.006	0.001
△HCS	**−0.444** ***	0.003	−0.005	0.031	−0.006	0.000	0.004	0.000
△WHO-5	**−0.343** ***	0.057	0.104	−0.182	−0.360	0.015	0.576	0.046
△PSQI - Total	**0.347** ***	0.026	0.003	−0.017	0.060	−0.008	0.089	0.019
△IDSS - Overall	**−0.785** ***	**0.010** *	−0.009	0.088	**0.043** *	0.001	**−0.058** *	0.005
DTSQc^e^	−0.010	**0.128** *	−0.120	−1.622	0.227	−0.105**	−0.247	0.076
△SUS	**−0.877** ***	0.102	−0.283	0.815	0.552	−0.115	−0.487	0.117

Data are estimated coefficient (β)

* p < 0.05

** p < 0.01

*** p < 0.001.

**Abbreviations:** ST, standard therapy; TBR, time below range; TIR, time in range; T1-DDS, Type 1 Diabetes Distress Scale; HCS, Hypoglycemia Confidence Scale; WHO-5, World Health Organization Well-Being Index 5; PSQI, Pittsburgh Sleep Quality Index; IDSS, Insulin Delivery System Satisfaction; DTSQc, Diabetes Treatment Satisfaction Questionnaire – change; SUS, System Usability Scale.

a For consistency in interpreting the regression analysis, the change for each questionnaire was calculated in such a way that a positive change indicates an improvement: baseline subtracted from follow-up for questionnaires where a higher score represents an improvement (HCS, WHO-5, IDSS, SUS), and follow-up subtracted from baseline for questionnaires where a lower score represents an improvement (T1-DDS, PSQI).

b Sex was coded such that male was set to 0 and female was set to 1.

c Change in percent TBR is calculated as follow-up minus baseline. A negative coefficient means that a larger (more negative) decrease in TBR was associated with a greater improvement in the questionnaire score outcome.

d Change in percent TIR is calculated as follow-up minus baseline. A positive coefficient means that a greater increase in TIR was associated with a greater improvement in the questionnaire score outcome.

e The DTSQs was used as the baseline questionnaire score for this regression analysis.

## Abbreviations:

ADA: American Diabetes Association
AID: Automated Insulin Delivery
CGM: Continuous Glucose Monitor
CSII: Continuous Subcutaneous Insulin Infusion
DTSQ: Diabetes Treatment Satisfaction Questionnaire
HCS: Hypoglycemia Confidence Scale
IDSS: Insulin Device Satisfaction Survey
MDI: Multiple Daily Injections
PSQI: Pittsburgh Sleep Quality Index
QOL: Quality of Life
SUS: System Usability Scale
ST: Standard Therapy
TIR: Time in Range
TBR: Time Below Range
T1D: Type 1 Diabetes
T1-DDS: Type 1 Diabetes Distress Scale
WHO-5: World Health Organization 5 Well-Being Index
