# Supplementary material for: How introduction of automated insulin delivery systems may influence psychosocial outcomes in adults with type 1 diabetes: Findings from the first investigation with the Omnipod® 5 System
Source: Diabetes Res Clin Pract. Author manuscript; Available in PMC 2024 Feb 28. (PMC10901155; doi:10.1016/j.diabres.2022.109998)
Supplement: 1 [file NIHMS1960240-supplement-1.docx]

# Online-Only Supplement

Table S1. Comparison of differences in psychosocial outcomes between prior standard therapies (MDI, tubed pump, or tubeless pump)

| Questionnaire | MDI | | Tubed | | Tubeless | | Mean  Difference  ± SEM  (Tubeless - MDI) | P-value^a^ | Mean  Difference  ± SEM  (Tubed - MDI) | P-value^a^ | Mean  Difference  ± SEM  (Tubed - Tubeless) | P-value^a^ |
| --- | --- | --- | --- | --- | --- | --- | --- | --- | --- | --- | --- | --- |
|  | **N** | **Mean**  **Change** | **N** | **Mean**  **Change** | **N** | **Mean**  **Change** |  |  |  |  |  |  |
| T1-DDS | 18 | -0.20 | 35 | -0.08 | 62 | -0.20 | 0.00 ± 0.10 | 0.9674 | 0.11 ± 0.11 | 0.3062 | 0.12 ± 0.09 | 0.1855 |
| HCS | 18 | 0.15 | 35 | 0.09 | 62 | 0.14 | -0.01 ± 0.10 | 0.9450 | -0.06 ± 0.09 | 0.5227 | -0.05 ± 0.08 | 0.5003 |
| WHO-5 | 18 | -1.11 | 35 | -0.91 | 58 | 0.41 | 1.5 ± 3.7 | 0.6834 | 0.20 ± 3.7 | 0.9575 | -1.3 ± 3.1 | 0.6699 |
| PSQI | 15 | -0.60 | 24 | 0.17 | 52 | -0.35 | 0.25 ± 0.58 | 0.6648 | 0.77 ± 0.78 | 0.3333 | 0.51 ± 0.54 | 0.3486 |
| IDSS | 18 | 0.18 | 35 | 0.21 | 62 | 0.13 | -0.04 ± 0.19 | 0.8155 | 0.04 ± 0.22 | 0.8728 | 0.08 ± 0.13 | 0.5498 |
| DTSQc^b^ | 18 | 14.89 | 35 | 10.97 | 61 | 12.87 | -2.0 ± 1.7 | 0.2509 | -3.9 ± 2.0 | 0.0607 | -1.9 ± 1.6 | 0.2293 |
| SUS | 16 | 13.59 | 33 | 9.02 | 62 | 5.77 | -7.8 ± 5.7 | 0.1743 | -4.6 ± 6.7 | 0.4954 | 3.2 ± 4.7 | 0.4917 |

^a^ Difference between indicated groups by two-sided t-test

^b^ The DTSQc score is assessed as a change from 0.0 (no change in treatment satisfaction)

**Abbreviations:** MDI, multiple daily injections; SEM, standard error of the mean; T1-DDS, Type 1 Diabetes Distress Scale; HCS, Hypoglycemia Confidence Scale; WHO-5, World Health Organization Well-Being Index 5; PSQI, Pittsburgh Sleep Quality Index; IDSS, Insulin Delivery System Satisfaction; DTSQc, Diabetes Treatment Satisfaction Questionnaire – change; SUS, System Usability Scale

Table S2. Comparison of differences in psychosocial outcomes between baseline HbA1c categories (<7% or ≥7% [53 mmol/mol])

| Questionnaire | HbA1c ≥7% [53 mmol/mol] | | HbA1c <7% [53 mmol/mol] | | Mean Difference  ± SEM  (HbA1c<7% - HbA1c≥7%  [53 mmol/mol]) | P-value^a^ |
| --- | --- | --- | --- | --- | --- | --- |
|  | **N** | **Mean**  **Change** | **N** | **Mean**  **Change** |  |  |
| T1-DDS | 61 | -0.18 | 54 | -0.14 | 0.04 ± 0.07 | 0.6021 |
| HCS | 61 | 0.17 | 54 | 0.08 | -0.09 ± 0.07 | 0.1958 |
| WHO-5 | 58 | 1.86 | 53 | -2.57 | -4.4 ± 2.6 | 0.0900 |
| PSQI | 49 | -0.37 | 42 | -0.12 | 0.25 ± 0.46 | 0.5871 |
| IDSS | 61 | 0.15 | 54 | 0.18 | 0.03 ± 0.13 | 0.8434 |
| DTSQc^b^ | 60 | 13.80 | 54 | 11.28 | -2.5 ± 1.3 | 0.0570 |
| SUS | 59 | 10.64 | 52 | 4.71 | -5.9 ± 4.0 | 0.1449 |

^a^ Difference between the two baseline HbA1c category groups as evaluated by two-sided t-test

^b^ The DTSQc score is assessed as a change from 0.0 (no change in treatment satisfaction)

**Abbreviations:** HbA1c, hemoglobin A1c; SEM, standard error of the mean; T1-DDS, Type 1 Diabetes Distress Scale; HCS, Hypoglycemia Confidence Scale; WHO-5, World Health Organization Well-Being Index 5; PSQI, Pittsburgh Sleep Quality Index; IDSS, Insulin Delivery System Satisfaction; DTSQc, Diabetes Treatment Satisfaction Questionnaire – change; SUS, System Usability Scale
