# Supplementary figures and images for: How introduction of automated insulin delivery systems may influence psychosocial outcomes in adults with type 1 diabetes: Findings from the first investigation with the Omnipod® 5 System
Source: Diabetes Res Clin Pract. Author manuscript; Available in PMC 2024 Feb 28. (PMC10901155; doi:10.1016/j.diabres.2022.109998)

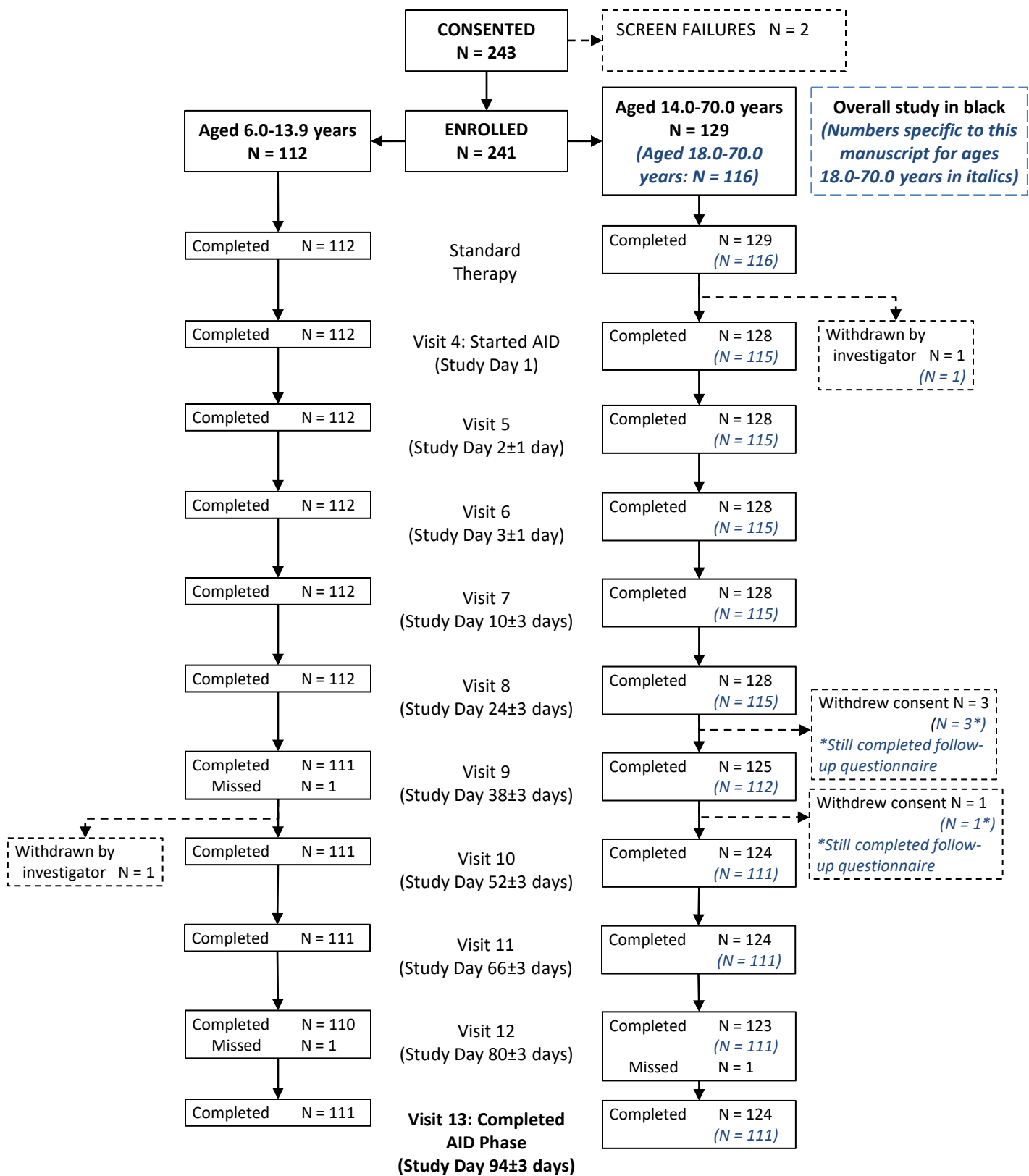

Supplement: 2 [file NIHMS1960240-supplement-2.pdf]
