# Supplementary material for: How introduction of automated insulin delivery systems may influence psychosocial outcomes in adults with type 1 diabetes: Findings from the first investigation with the Omnipod® 5 System
Source: Diabetes Res Clin Pract. Author manuscript; Available in PMC 2024 Feb 28. (PMC10901155; doi:10.1016/j.diabres.2022.109998)
Supplement: 3 [file NIHMS1960240-supplement-3.pdf]

CONSORT 2010 checklist of information to include when reporting a randomised trial*

Section/Topic	Item No	Checklist item	Reported on page No
**Title and abstract**			
	1a	Identification as a randomised trial in the title	N/A
	1b	Structured summary of trial design, methods, results, and conclusions (for specific guidance see CONSORT for abstracts)	7
**Introduction**			
Background and objectives	2a	Scientific background and explanation of rationale	8-9
	2b	Specific objectives or hypotheses	9
**Methods**			
Trial design	3a	Description of trial design (such as parallel, factorial) including allocation ratio	9-10†
	3b	Important changes to methods after trial commencement (such as eligibility criteria), with reasons	9-10†
Participants	4a	Eligibility criteria for participants	9†
	4b	Settings and locations where the data were collected	9, 22-24†
Interventions	5	The interventions for each group with sufficient details to allow replication, including how and when they were actually administered	9-10†
Outcomes	6a	Completely defined pre-specified primary and secondary outcome measures, including how and when they were assessed	11 µ
	6b	Any changes to trial outcomes after the trial commenced, with reasons	N/A
Sample size	7a	How sample size was determined	10†
	7b	When applicable, explanation of any interim analyses and stopping guidelines	N/A
Randomisation:			
Sequence generation	8a	Method used to generate the random allocation sequence	N/A
	8b	Type of randomisation; details of any restriction (such as blocking and block size)	N/A
Allocation concealment mechanism	9	Mechanism used to implement the random allocation sequence (such as sequentially numbered containers), describing any steps taken to conceal the sequence until interventions were assigned	N/A
Implementation	10	Who generated the random allocation sequence, who enrolled participants, and who assigned participants to interventions	N/A

Blinding	11a	If done, who was blinded after assignment to interventions (for example, participants, care providers, those assessing outcomes) and how	N/A
	11b	If relevant, description of the similarity of interventions	N/A
Statistical methods	12a	Statistical methods used to compare groups for primary and secondary outcomes	14-15 $^{\mu}$
	12b	Methods for additional analyses, such as subgroup analyses and adjusted analyses	14-15
**Results**			
Participant flow (a diagram is strongly recommended)	13a	For each group, the numbers of participants who were randomly assigned, received intended treatment, and were analysed for the primary outcome	15 $^{\mu}$
	13b	For each group, losses and exclusions after randomisation, together with reasons	16
Recruitment	14a	Dates defining the periods of recruitment and follow-up	10†
	14b	Why the trial ended or was stopped	N/A
Baseline data	15	A table showing baseline demographic and clinical characteristics for each group	29
Numbers analysed	16	For each group, number of participants (denominator) included in each analysis and whether the analysis was by original assigned groups	15, 29-35
Outcomes and estimation	17a	For each primary and secondary outcome, results for each group, and the estimated effect size and its precision (such as 95% confidence interval)	16-17, 31
	17b	For binary outcomes, presentation of both absolute and relative effect sizes is recommended	N/A
Ancillary analyses	18	Results of any other analyses performed, including subgroup analyses and adjusted analyses, distinguishing pre-specified from exploratory	15-17, 31-35, Supplement $^{\mu}$
Harms	19	All important harms or unintended effects in each group (for specific guidance see CONSORT for harms)	N/A†
**Discussion**			
Limitations	20	Trial limitations, addressing sources of potential bias, imprecision, and, if relevant, multiplicity of analyses	20-21
Generalisability	21	Generalisability (external validity, applicability) of the trial findings	19-21
Interpretation	22	Interpretation consistent with results, balancing benefits and harms, and considering other relevant evidence	18-21
**Other information**			
Registration	23	Registration number and name of trial registry	3, 7, 10
Protocol	24	Where the full trial protocol can be accessed, if available	N/A†
Funding	25	Sources of funding and other support (such as supply of drugs), role of funders	7, 25

*We strongly recommend reading this statement in conjunction with the CONSORT 2010 Explanation and Elaboration for important clarifications on all the items. If relevant, we also recommend reading CONSORT extensions for cluster randomised trials, non-inferiority and equivalence trials, non-pharmacological treatments, herbal interventions, and pragmatic trials. Additional extensions are forthcoming: for those and for up to date references relevant to this checklist, see www.consort-statement.org.

[µ] Please note: The study described in this manuscript was not a randomized control trial, so some items on this checklist are not applicable. The results in this study were not prespecified and were considered exploratory analyses.

[†] Since the manuscript for this psychosocial outcomes study describes a subset of data from a larger clinical study, more details are reported in another publication: Brown SA, Forlenza GP, et al. Multicenter Trial of a Tubeless, On-Body Automated Insulin Delivery System With Customizable Glycemic Targets in Pediatric and Adult Participants With Type 1 Diabetes. Diabetes Care. 2021; 44(7):1630-40. doi: https://doi.org/10.2337/dc21-0172

Items to include when reporting a randomized trial in a journal or conference abstract

Item	Description	Reported on line number
Title	Identification of the study as randomized	N/A
Authors *	Contact details for the corresponding author	
Trial design	Description of the trial design (e.g. parallel, cluster, non-inferiority)	3
Methods		
Participants	Eligibility criteria for participants and the settings where the data were collected	3-5
Interventions	Interventions intended for each group	5-8
Objective	Specific objective or hypothesis	1-2
Outcome	Clearly defined primary outcome for this report	N/A**
Randomization	How participants were allocated to interventions	N/A
Blinding (masking)	Whether or not participants, care givers, and those assessing the outcomes were blinded to group assignment	N/A
Results		
Numbers randomized	Number of participants randomized to each group	4
Recruitment	Trial status	4
Numbers analysed	Number of participants analysed in each group	4
Outcome	For the primary outcome, a result for each group and the estimated effect size and its precision	10-13**
Harms	Important adverse events or side effects	N/A
Conclusions	General interpretation of the results	17-18
Trial registration	Registration number and name of trial register	21
Funding	Source of funding	22

**this item is specific to conference abstracts*

**As this study was exploratory, no primary outcome was pre-specified.

Table 1. Information for Reporting Randomized Controlled Trials With Patient reported Outcomes

Section/Topic	Item	CONSORT 2010 Statement Checklist Item	PRO-Specific Extensions Are Prefaced by the letter P	Reported on Page No
		**Title and Abstract**		
	1a	Identification as a randomized trial in the title		
	1b	Structured summary of trial design, methods, results, and conclusions (for specific guidance see CONSORT for abstracts)[3]	P1b: The PRO should be identified in the abstract as a primary or secondary outcome	7*
		**Introduction**		
Background and objectives	2a	Scientific background and explanation of rationale	Including background and rationale for PRO assessment	
	2b	Specific objectives or hypotheses	P2b: The PRO hypothesis should be stated and relevant domains identified, if applicable	8-9
		**Methods**		
Trial design	3a	Description of trial design (such as parallel, factorial), including allocation ratio		
	3b	Important changes to methods after trial commencement (such as eligibility criteria), with reasons		
Participants	4a	Eligibility criteria for participants	Not PRO-specific, unless the PROs were used in eligibility or stratification criteria	
	4b	Settings and locations where the data were collected		
Interventions	5	The interventions for each group with sufficient details to allow replication, including how and when they were actually administered		
Outcomes	6a	Completely defined prespecified primary and secondary outcome measures, including how and when they were assessed	P6a: Evidence of PRO instrument validity and reliability should be provided or cited if available including the person completing the PRO and methods of data collection (paper, telephone, electronic, other)	11-14
	6b	Any changes to trial outcomes after the trial commenced, with reasons		
Sample size	7a	How sample size was determined	Not required for PRO unless it is a primary study outcome	
	7b	When applicable, explanation of any interim analyses and stopping guidelines		
		**Randomization**		
Sequence generation	8a	Method used to generate the random allocation sequence		
	8b	Type of randomization; details of any restriction (such as blocking and block size)		
Allocation concealment mechanism	9	Mechanism used to implement the random allocation sequence (such as sequentially numbered containers), describing any steps taken to conceal the sequence until interventions were assigned		
Implementation	10	Who generated the random allocation sequence, who enrolled participants, and who assigned participants to interventions		
Blinding	11a	If done, who was blinded after assignment to interventions (for example, participants, care providers, those assessing outcomes) and how		
	11b	If relevant, description of the similarity of interventions		
Statistical methods	12a	Statistical methods used to compare groups for primary and secondary outcomes	P12a: Statistical approaches for dealing with missing data are explicitly stated	14-15 *
	12b	Methods for additional analyses, such as subgroup analyses and adjusted analyses		
		**Results**		
Participant flow (a diagram is strongly recommended)	13a	For each group, the numbers of participants who were randomly assigned, received intended treatment, and were analyzed for the primary outcome	The number of PRO outcome data at baseline and at subsequent time points should be made transparent	15, 29
	13b	For each group, losses and exclusions after randomization, together with reasons		
Recruitment	14a	Dates defining the periods of recruitment and follow-up		
	14b	Why the trial ended or was stopped		
Baseline data	15	A table showing baseline demographic and clinical characteristics for each group	Including baseline PRO data when collected	15, 29, 31
Numbers analyzed	16	For each group, number of participants (denominator) included in each analysis and whether the analysis was by original assigned groups	Required for PRO results	31
Outcomes and estimation	17a	For each primary and secondary outcome, results for each group, the estimated effect size, and its precision (such as 95% confidence interval)	For multidimensional PRO results from each domain and time point	16-17, 31
	17b	For binary outcomes, presentation of both absolute and relative effect sizes is recommended		
Ancillary analyses	18	Results of any other analyses performed, including subgroup analyses and adjusted analyses, distinguishing prespecified from exploratory	Including PRO analyses, where relevant	17, Supplement
Harms	19	All important harms or unintended effects in each group (for specific guidance see CONSORT for harms)		
		**Discussion**		
Limitations	20	Trial limitations, addressing sources of potential bias, imprecision, and, if relevant, multiplicity of analyses	P20/21: PRO–specific limitations and implications for generalizability and clinical practice	18-21
Generalizability	21	Generalizability (external validity, applicability) of the trial findings		
Interpretation	22	Interpretation consistent with results, balancing benefits and harms, and considering other relevant evidence	PRO data should be interpreted in relation to clinical outcomes including survival data, where relevant	17-21
		**Other Information**		
Registration	23	Registration number and name of trial registry		
Protocol	24	Where the full trial protocol can be accessed, if available		
Funding	25	Sources of funding and other support (such as supply of drugs), role of funders		

Reproduced from *Reporting of Patient-Reported outcomes in Randomized Trials: The CONSORT PRO Extension. JAMA, February 27, 2013_Vol309(8)*

Copyright © 2013 American Medical association. All rights reserved."

*This study was exploratory and only contained post-hoc analyses.
